# Integration and Application of Multimodal Measurement Techniques: Relevance of Photogrammetry to Orthodontics

**DOI:** 10.3390/s21238026

**Published:** 2021-12-01

**Authors:** Dariusz Pojda, Agnieszka Anna Tomaka, Leszek Luchowski, Michał Tarnawski

**Affiliations:** Institute of Theoretical and Applied Informatics, Polish Academy of Sciences, Bałtycka 5, 44-100 Gliwice, Poland; dpojda@iitis.pl (D.P.); ines@iitis.pl (A.A.T.); dr.tarnawski@gmail.com (M.T.)

**Keywords:** photogrammetry, 3D photography, image registration, 3D model of the face and teeth, anthropometry, digital dentistry

## Abstract

Multimodal imaging, including 3D modalities, is increasingly being applied in orthodontics, both as a diagnostic tool and especially for the design of intraoral appliances, where geometric accuracy is very important. Laser scanners and other precision 3D-imaging devices are expensive and cumbersome, which limits their use in medical practice. Photogrammetry, using ordinary 2D photographs or video recordings to create 3D imagery, offers a cheaper and more convenient alternative, replacing the specialised equipment with handy consumer cameras. The present study addresses the question of to what extent, and under what conditions, this technique can be an adequate replacement for the 3D scanner. The accuracy of simple surface reconstruction and of model embedding achieved with photogrammetry was verified against that obtained with a triangulating laser scanner. To roughly evaluate the impact of image imperfections on photogrammetric reconstruction, the photographs for photogrammetry were taken under various lighting conditions and were used either raw or with a blur-simulating defocus. Video footage was also tested as another 2D-imaging modality feeding data into photogrammetry. The results show the significant potential of photogrammetric techniques.

## 1. Introduction

Photography and art have long accustomed us to perceiving and evaluating the aesthetics of human faces in 2D. This effect is compounded by the advent of the smartphone with its camera and social media connection, which makes people create and view more 2D pictures of themselves than ever before. More than once, a less-than-satisfactory 2D selfie has driven a person to seek aesthetic medical help.

One branch of medicine concerned with the appearance of the face as well as the correct functioning of the masticatory and articulatory systems is orthodontics.

Technological developments involving both cone-beam-computed-tomography (CBCT) imaging and 3D scanners have resulted in the introduction of the third dimension to orthodontic diagnosis.

Because of its nature, which uses radiation that passes through an object and that is absorbed differently in its various tissues, CBCT provides the ability to reproduce a three-dimensional model of a patient’s head composed of soft tissue models and a skeletal and dental model. Unfortunately, the use of invasive ionizing radiation greatly limits the use of this technique.

### 1.1. Conventional Approach: 3D Scans

3D scanners can image the outer surface of three-dimensional objects. They may use visible light, or near-infrared light, from ambient or dedicated light sources. Structured light sources such as lasers or light pattern projectors can also be used. Triangulation is the operating principle of many 3D scanners. These include both active solutions, which illuminate the object with invisible structured light patterns to provide the features required for 3D reconstruction, and passive ones, which reconstruct the scene directly from visual cues present in the acquired images. Other depth-acquisition techniques include: depth from defocus, depth from focus, pulse ranging, time-of-flight, phase-based, or continuous wave ranging methods [1], in addition to confocal, active wave sampling [2]. Types of 3D scanners relevant for orthodontic applications include (this list is by no means exhaustive):

- for face scanning: Vectra, 3DiD, 3dMD [3], EinScan Pro and Planmeca [4], Konica Minolta 910, and Polhemus [5];
- desktop scanners for scanning plaster dental models: Konica Minolta VI-9i and Imetric laser scanner [6];
- intraoral scanners: Trios, iTero Element, Dental Wings, Panda 2, Medit i500, Planmeca, and Aoralscan [7].

The applications of optical 3D facial scanners include a 3D analysis of facial features based on anatomical landmarks [8]; an analysis of facial symmetry [8,9]; a 3D analysis of facial deformations, as well as planning orthodontic treatment [10] and surgical interventions; and the evaluation of treatment results [11,12]. Statistical research is under way with the goal of developing anthropometric growth models of healthy children and models of faces of patients whose conditions lead to craniofacial deformations [13,14,15]. Growth models can be used in diagnostic systems for cases of cleft palate [15], fetal alcohol syndrome (FAS) [16] or DiGeorge syndrome, and others [14]. Facial scanners are increasingly being used in face-recognition systems [17], with results that allow a correct identification rate sometimes in excess of 99% [18].

3D scanning of plaster or polymer dental casts using dedicated scanners, or scanning dental arches directly with intraoral scanners, yields virtual dental models, which are a basis for dental analysis and for the design of various orthodontic appliances [10].

### 1.2. Multimodal Integration

The “conventional” orthodontic records include 3D dental models, 2D or 3D X-ray images, and photographs or 3D scans of the face, smile, and intraoral tissues. Such a body of information, in very diverse material representation and content, is already a multimodal set. Each of the imaging modes can be used in diagnostics on its own account, but bringing them together has a synergistic effect of creating a more-complete representation, display, and perception of the relevant structures [19]. In particular, by using a facial bow, some visible-light 3D images can be combined to embed the dental models within the 3D facial surface in the same position as actual teeth are situated within the face [20,21]. Such integration is relevant to medical records. The information obtained in this way from combining visible-light images is a simulation of what would otherwise require a CT scan, at the cost of a significant X-ray dose. The same technique of 3D-optical-image embedding can also be applied to the acquisition and analysis of mandibular motion [21].

The integrated data form a virtual model called the “virtual patient” [22], which can be used to increase the predicability and efficiency of dental treatment by virtual simulation of the treatment plan. An example of application of the embedding in prosthetics is the fabrication of complete dentures [23]. Other advantages of using this type of data representation include the ability to create high-precision anatomical documentation, to measure and evaluate clinical outcomes, and to improve communication with patients and with other professionals [24].

### 1.3. Accuracy: Requirements and Technically Achievable Levels

The virtual patient [22] is a simplified equivalent (image) of real anatomic structures of the patient. Therefore, the key problem of modelling is the degree of simplification, which is determined by the accuracy of the model. The aspect of model accuracy is important both from the point of view of how accurately the measuring device is able to measure the actual structures but also from the point of view of application—what accuracy is sufficient to ensure proper diagnosis, patient comfort, and the expected therapeutic effect of the appliances designed based on the model.

Doctors’ usual answer to the question about accuracy requirements was 1 mm for cephalometric analysis and 0.5 mm for plaster models, and these assumptions were checked when testing the implemented solutions [25,26]. Similar requirements for the accuracy of face scanning apply during intraoperative imaging [27], where a threshold below 1.0 mm was deemed acceptable and below 0.5 mm preferable. When specialists consult each other, it is estimated that verbal explanations of the geometry of the patient’s condition allows a mental image of it to be formed with an accuracy of about 2 mm.

The introduction of ISO 5725-1 [28] has unified the understanding of the concept of accuracy. The term “accuracy” is a combination of trueness and precision. Trueness (absolute accuracy) refers to the ability of the scanner to provide a 3D reconstruction as close to its true form as possible, and precision (repeatability accuracy) is the closeness of agreement between images acquired by repeated scanning procedures under the same conditions.

The last decade or so has seen a number of studies attempting to determine the accuracy of various types of scanners. Due to the lack of a calibration standard against which accuracies can be tested, the following approaches were used—either a reference object of known model is manufactured and then various measurements are taken [4,5,29] or the device with the highest nominal accuracy is used as a reference for the others [26,30].

According to a review article covering a very wide collection of studies analysing the accuracy of digital imaging of facial, skeletal, and intraoral tissues [31], the measured trueness for face scanners ranges from 0.140–1.380 mm and, for intraoral scanners, from 0.017–0.378 mm. In particular, the accuracy of face-mask scanning was about 0.10 mm [5] and of mannequin face scanning 0.52 mm [3]; for the 3dMD face scanner, the accuracy was 0.067 mm [32], while for the Konica Minolta Vivid 910 scanner, the accuracy was 0.08 mm [5]. Intraoral scanning achieves remarkable accuracy over small areas [7,33]. It performs less well over an entire dental arch [2,7]. Apparently, this is due to small registration errors accumulating over the series of single field-of-view local scans. In this respect, a desktop scanner examining a plaster or resin cast of teeth has the advantage of viewing the entire arch in a single view, without the need for multiple registration—as confirmed in comparative studies [6].

Since the integration of extra- and intraoral scans is not very widely practiced, there is relatively little research published on the accuracy of embeddings. According to two different publications, the accuracy attained is 0.78 mm [34] or 1.14 mm [30].

Many authors confirm that the 3D scanners available on the market have sufficient accuracy for the purposes of digital dentistry, refs. [2,7,31], but their cost can reach tens of thousands of EUR [29] both for facial and intraoral scanners [2].

### 1.4. Photogrammetry—Technical Principles and Applications

Photogrammetry is measuring based on photographs. It began in the mid-20th century as manual measurements and calculations based on large-format photographs taken with cameras meeting stringent geometric requirements to ensure undistorted projection [35]. With the image-processing capabilities offered by modern digital hardware, photogrammetry [36]—also known as multiple-view stereo [37]—is a rapidly evolving technique of deriving 3D information from multiple images taken from different, uncalibrated positions.

**Figure 1:**
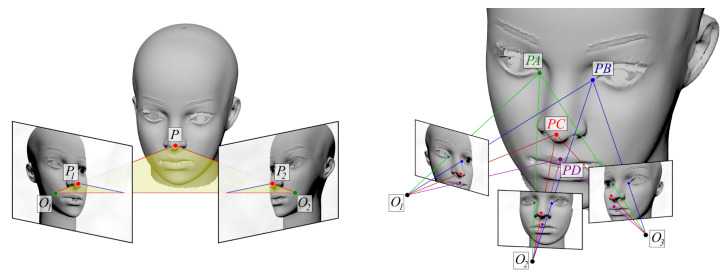
Idea of stereoscopic and photogrammetric reconstruction. In the case of stereoscopy (left drawing), rays starting at known optical centres of cameras, $O_{1}$ and $O_{2}$, and passing through image points, $P_{1}$ and $P_{2}$, intersect at 3D point *P*. In the right part of the picture, multiple photos from unknown camera positions are taken for photogrammetric analysis. Given intrinsic camera parameters, the distribution of points in each image determines a rigid bundle of rays originating from the camera. Photogrammetric reconstruction seeks to localise the cameras relative to each other so that all corresponding rays intersect. The intersection points are an estimate of actual 3D locations of scene features.

The principle of photogrammetry is essentially stereoscopic vision, i.e., reconstructing 3D coordinates of points from two or more 2D images. Every point of a 2D photograph represents a 3D point situated on a straight ray starting at the lens and pointed in a direction determined by the 2D coordinates. Its location along the ray is unknown, due to the loss of information in 3D-to-2D projection. Adding another photograph taken from a different viewpoint adds another ray and determines the 3D point as the intersection of the two rays (the left part of Figure 1). Applying this approach requires the position (location and orientation) of the cameras to be known, as well as their projection matrices relating 3D points in camera-centred coordinates to their projections in the image. All of those are determined during calibration. Formulas for stereoscopic 3D calibration and reconstruction can be found in [38,39,40].

While the stereoscopic approach requires the cameras to be precisely calibrated, photogrammetry (right part of Figure 1) bypasses the need for calibration by using a large number of photographs taken with the same camera and lens setting, which allows the system to self-calibrate and retrieve a 3D structure up to an unknown scaling factor [41]. Theoretically, the minimum number of photographs to achieve this is three. Corresponding rays from all cameras are constrained to pass through one point; this constraint is used to assess the mutual position of cameras and determine the position of scene points. Due to the finite resolution of cameras and optical imperfections, the constraints are met with finite accuracy. A greater number of photographs providing redundant information allows an optimization process—bundle adjustment [42]—to improve accuracy. The ambiguity of scale can be removed if at least one distance is measured or otherwise ascertained between any two points.

The mathematical derivation of photogrammetric reconstruction can be found in [40], where two approaches are discussed: the Kruppa equations and the use of the absolute conic—a circular construct consisting of points with complex coordinates situated on the plane at infinity, which is present in all images and can be used as a calibration pattern [40].

Various implementations of photogrammetric reconstruction are possible. The one included in the Alice Vision framework [43] requires the following steps:

- image segmentation—localizing feature points in each image. Features are not restrained to corners or line intersections; they can include small image areas identifiable between two or more images due to similarity measures such as correlation, cepstral transform [44], or Scale-Invariant Feature Transform (SIFT) [45]. In high-resolution images, small details of texture such as skin pores and hairs can be used.
- feature matching—identifying features in different images that represent the same feature in reality;
- autocalibration—using the matched features to identify the mutual relations between the camera positions the images were taken from;
- stereoscopic reconstruction—using the calibration data and 2D features from pairs or triplets of images to find the 3D coordinates; this step yields depth maps that constitute partial models of the scene as perceived from a certain direction.
- registration—bringing partial models into a common coordinate system;
- bundle adjustment—merging all the available information to integrate the model and improve its accuracy by optimizing for best compatibility with the image data. The result is a dense point cloud, which is triangulated next to produce a mesh surface model.

Some current face scanners use photographs of the patient’s face acquired from several viewpoints, obtained at the same moment in time. However, their cameras are placed either on an arm, a rig, or a frame [3]. This ensures their geometric relationships remain constant, and their earlier calibration is possible, which greatly simplifies computation and improves accuracy. Although the name “phogrammetry” is used, they actually function more as stereogrammetry or stereo-photogrammetry [3]. A similar working principle is also used by some intraoral scanners [2].

Some early work on orthodontic applications of uncalibrated photogrammetry can be found in [32] for face scanning, in which the deviation of smartphone-photos-based photogrammetry was about 0.604 mm versus 0.060 mm for Konica Minolta Scanning, and in [46] for plaster-model scanning, in which the discrepancy between plaster model-based and photogrametric reconstruction-based measurements ranged from 0.433 to 0. 611 mm.

### 1.5. The Goal of This Work

Three-dimensional scanners are still mainly available in university clinics and in a small proportion of private practices. Cheaper alternatives are therefore pursued, but not all of them meet the requirements of digital dentistry [27,29];

As an affordable system for 3D orthodontic imaging, photogrammetry is a plausible alternative, if adequate accuracy can be achieved.

The purpose of this work was to verify the accuracy of photogrammetric reconstructions, to establish what factors influence the accuracy level, and to evaluate the potential of photogrammetry as part of multimedial orthodontic applications.

## 2. Materials and Method

To achieve the aim of the work, answers to the following questions were sought:

1. To what extent do the working conditions of a photogrammetric system, such as the type of lighting and the camera settings, affect the quality of reconstruction?
2. What is the best attainable accuracy of photogrammetric 3D reconstruction for intraoral and extraoral imaging? and is the accuracy sufficient to replace professional 3D scanners?
3. What accuracy can be attained with the technique of embedding [20]? How does the embedding of intraoral models inside extraoral photogrammetric 3D reconstructions compare to the same embedding procedure performed with 3D scans?

### 2.1. Methods of Assessing Accuracy

To evaluate the usability of photogrammetry, an experiment was carried out, comparing photogrammetric reconstructions taken under different simulated impediments to 3D scans.

The results were compared in two ways: qualitatively (visually) and quantitatively—by measuring the distance between the photogrammetric models and the 3D scans taken as a reference.

All models were represented by triangular meshes. Comparing them requires a method of determining the correspondence between meshes and a measure of distance. If correspondence is unknown, the closest vertex of the opposite mesh, or the closest point on the closest face of the opposite mesh, is taken as the counterpart of each vertex. The Euclidean distance is used as a measure of closeness throughout.

Traditionally, according to [47,48], the measure of the distance between the surfaces can be defined as:

$$E_{m}\left(S,S^{\prime }\right)=\frac{1}{\left|S\right|}\int_{p\in S}e\left(p,S^{\prime }\right)ds$$

where the distance *e* of any point *p* of *S* to surface $S^{\prime }$ may be defined as the Euclidean distance between *p* and its counterpart $p^{\prime }$.

$$e\left(p,S^{\prime }\right)=\min_{p^{\prime }\in S^{\prime }}d\left(p,p^{\prime }\right)$$

The distance between meshes *M* and $M^{\prime }$ can be defined as a discrete version of the formula cited above, which is tantamount to determining the average Euclidean distance between the vertices and their counterparts.

$$E_{m}\left(M,M^{\prime }\right)=\frac{1}{n}\sum_{i=1}^{n}\min_{p_{i}^{\prime }\in M^{\prime }}d\left(p_{i},p_{i}^{\prime }\right)$$

To avoid the influence of unmatched parts, only vertices that do have a counterpart on the other mesh within a certain radius were taken into account. In all the evaluations of the accuracy of photogrammetric reconstructions, the average distance and the standard deviation were measured between the mesh obtained from photogrammetry and a 3D scan taken as a reference.

The distance between two meshes can be determined in any position of one mesh relative to the other. For our purposes, the relevant position was the one in which the distance is smallest. This position was found by the process of registration.

In the process of registration, the coordinates of counterparts are used to determine a transformation consisting of scaling, rotation, and translation, for which the sum of distances between counterpart points will be smallest.

This is achieved by the Iterative Closest Point (ICP) algorithm [49]). Its principle consists in iterating the following steps:

- determining the distance between meshes in their current position;
- determining the transformation that minimizes the distance;
- applying the transformation to one of the meshes.

The loop ends when the distance between the meshes has reached a minimum or when it is less than a preset threshold.

### 2.2. Factors Affecting Accuracy

At first, the best camera settings and lighting parameters for taking pictures for photogrammetric reconstruction were identified, and the results obtained by various kinds of photogrammetric software were compared. Photogrammetric reconstruction of faces was tested using a hairdresser’s dummy head with a realistic shape and colour.

The photographs were taken with a mid-market 16 MPix camera and a standard 14–42 mm lens. In some cases, additional elements were introduced in the background to assist in the process of image registration.

The sensitivity of photogrammetric reconstruction to imperfect imaging conditions was evaluated by deliberately blurring images and altering their brightness before submitting them to the software. The influence of image quality on stereogrammetric reconstruction was also studied in [50] in the context of histogram-based preprocessing.

After selecting the camera parameters and taking a series of photographs, reconstruction was performed using different existing software systems: Agisoft Metashape Professional, MeshRoom (AliceView), and ColMap + MVS.

### 2.3. Scanning Methods

Photogrammetric imaging can be applied wherever optical scanning is used, i.e., to acquire the shape and colour texture of the visible surfaces of body parts. In order to verify the applicability of photogrammetric techniques to scan faces and dental models and to integrate them for medical or similar purposes, the scanning method for each of them needs to be determined.

#### 2.3.1. Face-Scanning Methods

For face scanning, it is important to obtain a surface model with a quality that will allow selected anatomical features to be determined and located. The models must reproduce the surface of the patient’s skin with sufficient accuracy; on the other hand, some details like hair are less critical. The pictures taken must cover the whole face without gaps and with sufficient overlap.

The solution might be to use multi-camera rigs taking a number of photos at the same time. Its cost, however, may be comparable to the cost of a scanner.

A series of still pictures provides images of good quality and resolution. As the camera is moved between viewpoints, it is difficult for a human subject to remain motionless. So, the process should be as short as possible.

Another possibility is to use the camera in video-recording mode. Video sequences can be taken in less time, and they ensure good coverage and overlap between successive frames, which are only slightly shifted with respect to each other. On the other hand, the volume of data is overwhelming, and a subset of frames has to be selected. Motion blur is an additional problem. The solution can include reducing the exposure time or increasing the frame rate, using point autofocus or manual/semiautomatic removal of the blurred frames.

As 5 MPix is the minimum resolution recommended by Agisoft software, the 4K video format (3840 × 2160) was used in the present project, at 60 fps. To reduce the processing time, every tenth frame was taken, resulting in a series of about 400 images each. Still photographs were taken at 16 MPix resolution.

**Figure 2:**
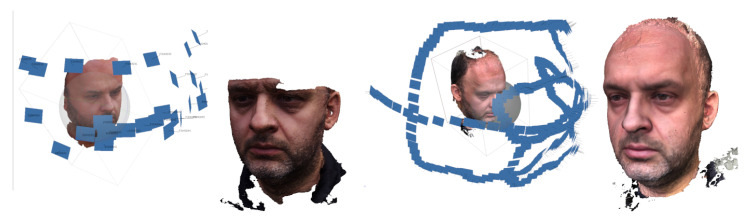
Reconstructing a subject’s face based on still photos and in video mode. Different sequences of camera positions while shooting and the resulting face models.

**Figure 3:**
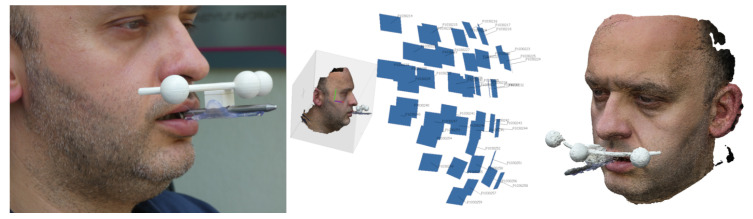
Scanning a subject’s face with facial bow. Camera positions while shooting and the reconstructed surface model.

Images were taken of a highly motivated adult subject, distinguished by patience and the ability to stay motionless (Figure 2). In this and some subsequent figures, small blue rectangles suspended in 3D space represent the camera positions. To evaluate the accuracy of the embedding method described in Section 2.4, an additional scan was performed of the face with the facial bow (Figure 3).

#### 2.3.2. Scanning Teeth or Dental Models

There are two methods to obtain a digital representation of the form of the patient’s dental arches. The indirect one is by taking dental impressions and using them to cast plaster (stone) models, then scanning those models. The direct method is to use intraoral scanners [51]. The primary drawback of such devices is the price, limiting their accessibility for small medical practices.

Dental casts are easy to photograph, as they are small, rigid, inanimate objects that can be placed on a rotating table in a variety of positions. It is thus of interest to examine the accuracy of their photogrammetric reconstruction.

When scanning plaster dental models, the useful information is limited to one side of the cast; the flat side can be left out. For photogrammetric reconstruction, one series of photographs taken on a rotating table is sufficient.

**Figure 4:**
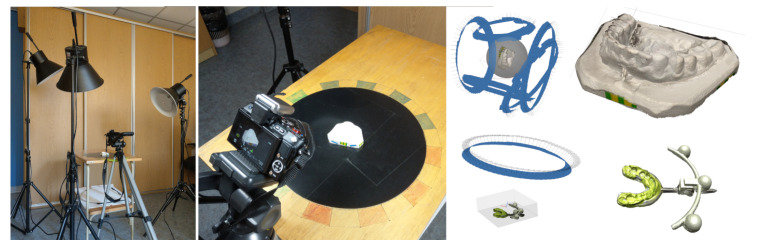
Left and centre: photographing a dental model. Right: camera positions while shooting and the reconstructed digital models.

For the embedding technique described in Section 2.4, a full digital representation of the cast was required, including the flat underside. Obtaining such a model requires reconstructions to be built for several positions of the cast on the rotating table and then integrated into one complete mesh (Figure 4).

An imaging station for casts built for the present work comprised a 16 Mpix camera, a rotary table turning in steps of $5^{\circ }$, and a set of lamps.

### 2.4. Model Embedding

The purpose of model embedding is to position a virtual model of the dental arches inside a virtual model of the face. “Embedding” will hereafter also refer to the result: a 3D image composed of the face with teeth inside.

The method [20] uses a facial bow to create a link between the internal and external geometry of the patient’s face. The following scans—or photogrammetric reconstructions—are used: of the face at rest; of dental models; of the face with facial bow inserted; and of dental models with the facial bow.

**Figure 5:**
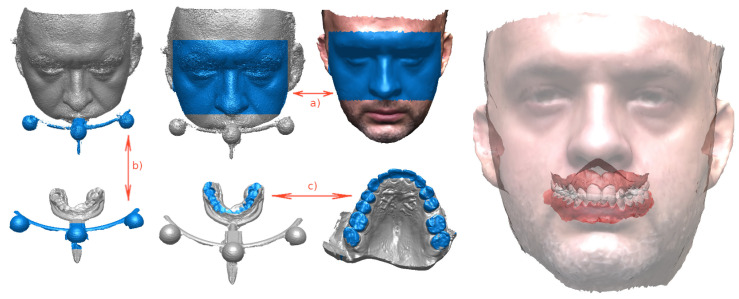
Using a facial bow to embed dental models in the face. The areas marked in blue, common to pairs of models, are used to bring the models into register. The upper part of the face (eyes and nose) is used to align the “face alone” scan with the “face and bow” (**a**). The external part of the bow is used to align “face and bow” with the dental models (**b**). Finally, the two sides of the bit attached to the bow and the matching occlusal surfaces of the patient’s teeth (**c**) allow the latter to be brought into register with all the models aligned so far.

This approach is based on finding the common parts between the various scanned surfaces (Figure 5). All of them should be brought into register by rigid-body transforms, identified by correlating the common parts.

The method was modified to accommodate photogrammetric reconstructions instead of 3D scans. As each photogrammetric reconstruction is obtained at a different scale, a flexible-scale version of the ICP algorithm should be used. The facial bow, designed and 3D printed with known dimensions, can be used as a calibration object.

Integrating the photographs from multiple rotary-table sessions under Agisoft proved problematic (Section 4). Only the relevant part of the dental cast was used, i.e., the model of the teeth, rejecting the flat undersides, which were extensively used for registration in previous work [52]. The model of the teeth was therefore aligned with a negative of the occlusal portion.

## 3. Results

The purpose of quantitative analysis is to assess the accuracy of photogrammetric reconstruction and to verify if its use in model embedding yields similar results as when using 3D scans. In either case, accuracy is evaluated by the error measure described in Point Section 2.1.

### 3.1. Comparing the Accuracy of Reconstruction from Image Series Taken under Different Conditions

**Figure 6:**
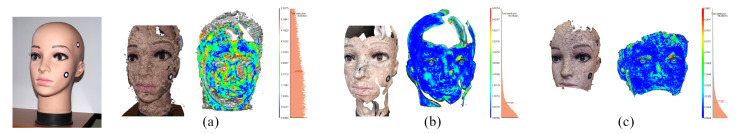
Reconstruction results from the three test series (Table 1).

The first trials were intended as a qualitative search for the best type of lighting, white balance, and camera support. By roughly analysing three series of photographs (Table 1), the best results were obtained (see Figure 6) for diffuse shadowless lighting with a manually set white balance.

The conditions under which the photographs were taken proved extremely important. The influence of lighting had a particularly great impact; it should be even and as shadowless as possible. Pictures taken with the use of flash were sharp, but the deep shadows visible in them were misinterpreted by software, resulting in undesirable artefacts.

More systematic research into the influence of lighting on accuracy was carried out using the following approach: Modifying a series of images rather than taking a new one under different conditions was used to assess the impact of stronger or weaker photographic exposure. Instead of taking multiple series of photographs, which would inevitably change many other aspects of the images besides exposure, a software program was used to virtually develop .raw image data into .jpg images, emulating various exposure-value (+−EV) settings of the camera. The procedure implemented in the software was the same as would have been used by firmware in the camera itself when modifying the EV settings.

**Figure 7:**
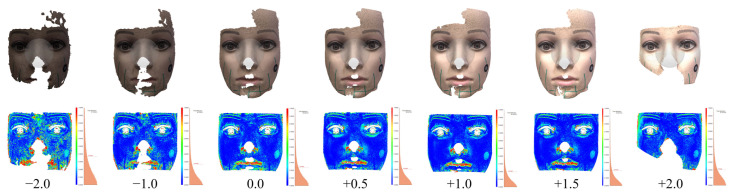
Evaluating reconstructions from series of images with various EV (Table 2).

The results obtained (Table 2, Figure 7) show that, for the given areas, increased brightness improved the accuracy of photogrammetric reconstructions, up to a certain point where saturation begins, which caused accuracy to deteriorate again. The reason for this is that changed exposure values brings out additional image features, which can be used for reconstruction. This is also confirmed by an analysis of the area of the cut-out reconstructed parts, which exemplifies the difficulties of reconstructing from underexposed as well as overexposed images.

**Figure 8:**
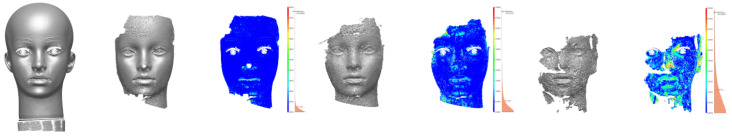
Evaluating reconstructions from the original series of images and with various degrees of blurring (Table 3).

To evaluate the influence of blurring on the quality of photogrammetric reconstruction, a series of photographs was deliberately blurred by a low-pass filter. This approach was chosen rather than defocussing the camera, because it ensured that all other aspects—except the focus—of the images would be unchanged. In fact, from a methodological point of view, the authors had to choose between two options: either use a whole population (at least in the order of tens) of series of photographs taken with a correctly focussed lens, and a similar population of series of photographs taken with the focus deliberately maladjusted by a certain degree, then compute the average reconstruction quality from either population and compare the two or use just one series with either lens setting, ensuring the camera positions and other parameters—beside the focus—were the same. The first approach would be prohibitively time consuming, and the second was unfeasible in the physical reality, which is why a “virtual defocus” (i.e., blurring by a low-pass filter) was chosen. The photogrammetric reconstructions were compared to laser scans acquired by the Konica Minolta scanner with a tele lens. Results presented in Figure 8 and Table 3 show that blurring corrupted the accuracy of photogrammetric reconstruction. Higher values of the blurring parameter σ led to poorer reconstruction qualities.

Photogrammetric reconstruction was carried out using three different packets of software: open-source Meshroom (based on AliceView), open-source ColMap combined with OpenMVS, and commercial Metashape.

**Figure 9:**
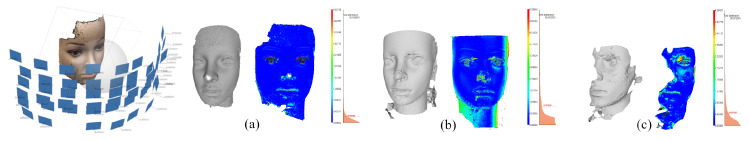
Comparison of photogrammetric reconstruction obtained from the same set of photographs (**left**) using Agisoft Metashape (**a**), MeshRoom (AliceView) (**b**), and ColMap + MVS (**c**) software. (Table 4).

The best reconstructions, for this set of data, were obtained from the Agisoft Metashape software. Figure 9 and Table 4 shows a comparison of the results. This software was used throughout the present work, which did not attempt to create new photogrammetric algorithms or improve upon existing ones.

### 3.2. Accuracy of Photogrammetry

To assess the quality of photogrammetric reconstruction, reconstructions were compared to 3D scans for the following objects: dental casts, a dummy face, and a live face. The results are shown in Table 5. The reconstruction quality was evaluated by analysing the distances between meshes after aligning them by the ICP algorithm.

**Figure 10:**
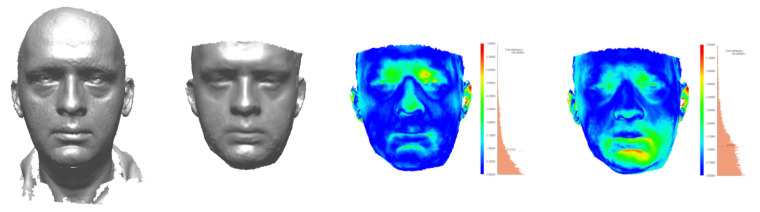
3dMD face scan, face photogrammetric reconstruction, and their ICP-based and bow-based registrations.

**Figure 11:**
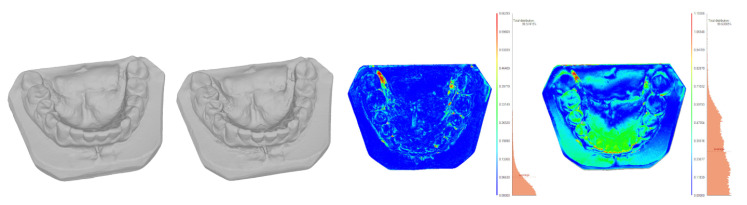
Minolta dental model scan, dental model photogrammetric reconstruction, and their ICP-based and bow-based registrations.

For inanimate objects, the deviation was of the order of 0.2 mm; for humans, it was 1 mm. To assess the impact of involuntary movements, distances were also measured between two 3dMD scans taken at a short time interval from each other, without a deliberate change in facial expression. The average distance was 0.39 mm, and the std. dev. was 0.43 mm. The results of comparisons are shown in Figure 9a for the dummy face, Figure 10 for the live face, and Figure 11 for the tooth models.

### 3.3. Comparing the Embeddings

To assess the accuracy of photogrammetric scanning under practical circumstances, the process of embedding (Figure 5) was performed twice: for photogrammetric reconstructions and for 3D scans.

In the first case, the registration steps used the CAD model of the facial bow, as designed prior to printing, and photogrammetric reconstructions of the following objects: the occlusal portion with the facial bow attached, the dental models, the face with the occlusal portion and the bow attached, and the undisturbed face.

In the second case, the same CAD model of the facial bow was combined with the 3D scans of the same objects.

In either case, all models were brought into register with the facial bow, resulting in an embedding of the virtual dental models in the face mesh, all in the same fixed coordinate system of the CAD-designed bow.

**Figure 12:**
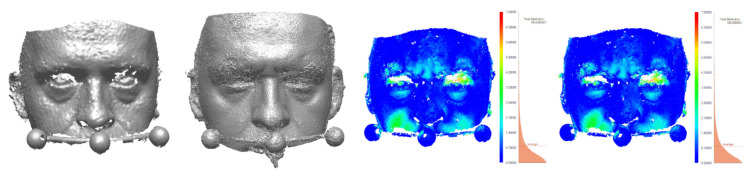
Left to right: scan of face and bow taken with the 3dMD, face-and-bow photogrammetric reconstruction, and their ICP-based and bow-based registrations with error distributions.

**Figure 13:**
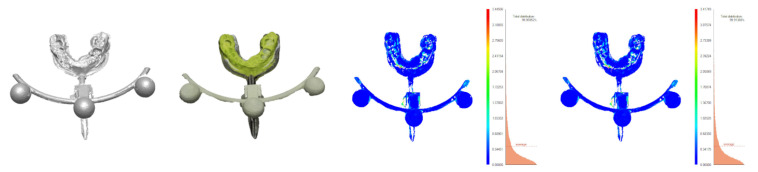
Scan of occlusal portion taken with the Minolta scanner, occlusal portion photogrammetric reconstruction, and their ICP-based and bow-based registrations.

Theoretically, the corresponding elements of either embedding should coincide. In practice, their positions differed, and the distances between them were evaluated (Columns 4 and 5 of Table 6). They result from a combination of factors, including imaging discrepancies (differences of shape) and the errors of the registration process (differences in position) Figure 10, Figure 11, Figure 12 and Figure 13).

To estimate the different components of the distance, each pair of corresponding models was also aligned directly, with the use of the ICP algorithm, bringing the two models of the same object as close as possible, without considering any other parts. The error of this alignment, i.e., the difference between the two meshes, is as an estimate of the discrepancy between the different imaging techniques: scanning and photogrammetry. They are presented in Figure 10, Figure 11, Figure 12 and Figure 13 and Columns 2 and 3 of Table 6.

The position error of facial bow-based alignment was represented as a rigid-body transform, i.e., a rotation and a translation from the position of each element (as shown is the rows of Table 6) in the embedding to the direct ICP-based registration of the element to its counterpart. These error values are shown in Columns 6 and 7 of Table 6.

Columns 2 and 3 represent the distances between the photogrammetric reconstruction and the scan of individual objects after ICP reconstruction; Columns 4 and 5—after bow-based registration. Columns 6 and 7 present the parameters of a rigid-body translation from bow-based embedding to individual ICP-based alignment of corresponding elements in photogrammetric and 3D-scan imagery. It can be noticed in Table 6 that the embedding error increases along the chain of registrations from the facial bow to the models of the face and the teeth. Apparently, errors propagate and accumulate in successive registrations. Both the imaging discrepancy and the registration error of photogrammetric reconstruction were smaller for rigid objects than for soft tissues.

To compare the embeddings achieved with the use of 3D scans and those based on photogrammetry, the two embeddings were aligned by their tooth models, and the distance between the two facial models was measured.

## 4. Discussion

The area covered by photogrammetric reconstruction strongly depends on how many of the photographs the software has been able to bring into register and calibrate.

From this point of view, fuller reconstructions were obtained from image sequences extracted and filtered from video footage. The coverage was better, and the massive redundancy makes the technique robust to the case when some of the frames cannot be registered correctly. The processing, however, can be very time-consuming, especially as acceptable accuracy can only be achieved with high-resolution video. Photogrammetric reconstruction from a series of still photographs is less costly in processing time, but failure to bring some pictures into register can result in an incomplete reconstruction.

**Figure 14:**
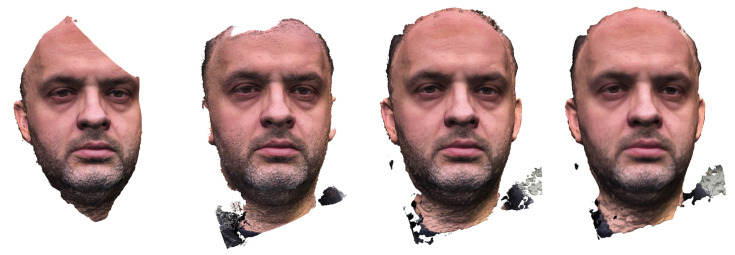
The number of images included in a reconstruction affects its extent.

Our observations indicate that the success of photogrammetric reconstruction depends on many conditions, such as lighting, stable position of the object, judicious selection of viewpoints, and elimination of blurred images. As the process relies on optimization, the choice of parameters has a significant impact on the quality of the results, including the inclusion or rejection of some images in the reconstruction (Figure 14).

The quality of photogrammetric reconstruction is influenced by factors such as type of lighting, the number of photographs taken—which results in better or poorer coverage—and the quality of the photographs. The latter depends on the class of the camera and lens but also on the kind and complexity of the surface.

The observed dependence of the quality of photogrammetric reconstruction on lighting, 2D image quality, and the distribution of viewpoints suggests the need for further systematic studies for many patients, and for many device configurations. Such studies are beyond the scope of the present work. They could lead to the development of an accurate model of the impact of image capture conditions on the accuracy of reconstruction, to the identification of optimal conditions for image acquisition, and to specifying when further improving the image quality is no longer worthwile in terms of the cost vs effect tradeoff. Pending more precise findings, the images should be taken of a calmly seated patient, with a good lighting which allows camera diaphragm to be narrowed, increasing the depth of focus. In the controlled conditions of a medical facility, this should be readily feasible.

In the case of a human subject, his/her patience is also important. Photographing children is especially problematic, as their attention span is often too short for a full series of photographs. This constraint may be alleviated in the future, with the development of nonrigid photogrammetry.

Mimic or respiratory movements are less of a problem in intraoral scanning, an important tool in orthodontics. Many existing solutions use a 2D intraoral camera and photogrammetric algorithms. The areas of greatest interest, i.e., the teeth and gums, can be considered rigid during the time of scanning.

**Figure 15:**
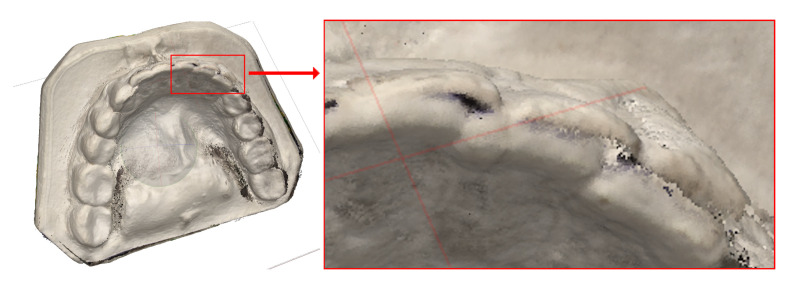
Reconstruction. Enlarged fragment shows registration error: the unnatural split in the incisor edges, which is due to a scale difference between the reconstructed surfaces on the lingual and the labial side.

As photogrammetry is liable to scale errors, it can lead to problems exemplified by Figure 15. To avoid this effect, calibrating objects should be used, or photogrammetric imagery supplemented by other imaging modalities.

In spite of its limitations, photogrammetry can be useful for the purposes of medical documentation where more advanced imaging techniques are not available. Especially at the times of the COVID-19 pandemic, when the referring primary care doctor and the patient may not immediately be in personal contact with the specialist, the referral would otherwise need to include a verbal description. At little or no additional cost, photogrammetric reconstruction is more accurate by a factor of 2 (an accuracy of 1 or 1.2 mm against 2 mm for verbal description). The primary care doctor may just send judiciously taken photographs (of the plaster models or the face with the facial bow), which the specialist will use in his office to form a photogrammetric reconstruction. Photographs can even be taken by the patient in their home.

The taking of photographs or videos for the purposes of photogrammetry can be assisted by dedicated smartphone application programs and cheap simple mechanical/optical aids such as the ScanBox by Dental Monitoring [53], which provides cheek retraction and mechanical positioning and a slide for the smartphone to move along, while the application program provides verbal instructions for the patient filming their own teeth at home. Similar aids and procedures can be developed for primary medical practices.

Photogrammetry is developing rapidly, benefiting from progress both in computing hardware and in algorithms. Its accuracy should improve dramatically in the near future. Medical practitioners may be well advised to store photographs and video footage for documentation and for future statistical analysis, contributing to the development of standards and diagnostic criteria.

## 5. Conclusions

- Photogrammetry can be used in digital dentistry;
- Its accuracy is satisfactory for documentation purposes, for medical reference, as well as in case of any mishap and compensation or liability procedures;
- It constitutes a much cheaper alternative to laser and other 3D scanners;
- As both algorithms and cameras develop, it may become adequate for most applications other than those mentioned above, e.g., for the digital design and 3D printing of dental appliances
- As accuracy of reconstruction is likely to improve with subsequent generations of photogrammetric software, medical practitioners would be well advised to save large sets of photographs for future processing and research.

## Figures and Tables

**Table 1:** Conditions while taking photographs (Figure 6) and the accuracy achieved.

Series	Number of Photos	Lighting Daylight	f (mm)	Expos. Time (s)	Cam. Mount	White Bal.	Avg Dist (mm)	Std. Dev. (mm)
(a)	16	+flash	14	1/160 man	freehand	auto	2.464	1.602
(b)	27	+fluors.	22	∼1/10 auto	tripod	auto	0.776	0.939
(c)	22	+fluors.	42	∼1/20 auto	tripod	man	0.738	0.852

**Table 2:** Accuracy of photogrammetric reconstruction for different photographic exposure (Figure 7).

EV	Compared to Minolta, Trimmed		Surface
	Dist (mm)	Std Dev (mm)	Area $\left({\mathrm{cm}}^{2}\right)$
2.0	0.202	0.31	161.56
1.5	0.195	0.3	213.7
1.0	0.197	0.35	213.19
0.5	0.204	0.34	214.96
0.0	0.237	0.369	209.28
−1.0	0.269	0.424	207.56
−2.0	0.368	0.496	198.61

**Table 3:** Accuracy of photogrammetric reconstruction for different degrees of blurring (Figure 8).

1	2	3
	ICP	ICP
3D surfaces	avg	stdev.
comparison	(mm)	(mm)
No blurring	0.21	0.36
30	0.49	0.67
60	1.07	1.02

**Table 4:** Accuracy of photogrammetric reconstruction from the same set of images with various software (Figure 9).

	Used	Avg Dist	Std. Dev.
	Software	(mm)	(mm)
(a)	Agisoft Metashape	0.212	0.365
(b)	MeshRoom	0.868	1.181
(c)	ColMap + MVS	0.441	0.644

**Table 5:** Accuracy of photogrammetric reconstructions of (Figure 9a, Figure 10 and Figure 11).

Object		Photogramm. to Scan		Scan to Scan	
		Dist.	Std. Dev.	Dist.	Std. Dev.
		(mm)	(mm)	(mm)	(mm)
Dental models		0.22	0.95		
Dummy face		0.21	0.36		
Live face	Stills	1.16	1.18	0.39	0.43
	Video	1.03	1.00		

**Table 6:** Quantitative comparison of ICP-based vs. bow-based registrations.

1	2	3	4	5	6	7
**3D**	**ICP**	**ICP**	**Bow**	**Bow**	**Rot.**	
**Surfaces**	**Avg**	**Std. Dev.**	**Avg**	**Std. Dev.**	**Angle**	**disp.**
**Comparison**	**(mm)**	**(mm)**	**(mm)**	**(mm)**	**(^∘^)**	**(mm)**
Face+bow	0.58	0.64	0.58	0.65	0.13	0.11
Face	1.03	1.00	1.37	1.13	1.83	1.26
Bit	0.42	0.87	0.45	0.94	0.01	0.01
Teeth	0.22	0.95	0.44	0.95	0.46	0.51
Embedding	1.03	1.00	1.53	1.21	1.24	1.98

## Data Availability

The data presented in this study are available, on a need-to-know basisis, on request from the corresponding author. The data are not publicly available due to the sensitive nature of the medical imagery of dental arches, often affected by developmental faults or other deformities. Anonymization is a dubious solution in the case of facial images.
